# Effect of Ageing on the Mechanical Properties of Dental Resin with and Without Bisphenol A

**DOI:** 10.3390/ma18122704

**Published:** 2025-06-09

**Authors:** Lígia Lopes-Rocha, Orlanda Torres, Joana Garcez, Ricardo J. C. Carbas, Catarina Borges, Vírginia M. F. Gonçalves, Maria Elizabeth Tiritan, Igor Studart Medeiros, Teresa Pinho, Lucas F. M. da Silva

**Affiliations:** 1 UNIPRO—Oral Pathology and Rehabilitation Research Unit, University Institute of Health Sciences (IUCS-CESPU), 4585-116 Gandra, Portugal; orlanda.torres@iucs.cespu.pt (O.T.); joanagarcez@hotmail.com (J.G.); teresa.pinho@iucs.cespu.pt (T.P.); 2 Department of Mechanical Engineering, Faculty of Engineering (FEUP), University of Porto, Dr. Roberto Frias Street, 4200-465 Porto, Portugal; rcarbas@fe.up.pt (R.J.C.C.); lucas@fe.up.pt (L.F.M.d.S.); 3 Institute of Science and Innovation in Mechanical and Industrial Engineering (INEGI), Dr. Roberto Frias Street, 4200-465 Porto, Portugal; cborges@inegi.up.pt; 4 Associate Laboratory i4HB, Institute for Health and Bioeconomy, University Institute of Health Sciences, CESPU, 4585-116 Gandra, Portugal; virginia.goncalves@cespu.pt (V.M.F.G.); elizabeth.tiritan@iucs.cespu.pt (M.E.T.); 5 UCIBIO—Applied Molecular Biosciences Unit, Translational Toxicology Research Laboratory, University Institute of Health Sciences (1H-TOXRUN, IUCS-CESPU), 4585-116 Gandra, Portugal; 6 The Laboratory of Organic and Pharmaceutical Chemistry (LQOF), Department of Chemical Sciences, Faculty of Pharmacy, University of Porto, R. Jorge de Viterbo Ferreira 228, 4050-313 Porto, Portugal; 7 Interdisciplinary Center of Marine and Environmental Research (CIIMAR), Edifício do Terminal de Cruzeiros do Porto de Leixões, Av. General Norton de Matos s/n, R. Jorge de Viterbo Ferreira 228, 4050-313 Porto, Portugal; 8 Biomaterials and Oral Biology Department, School of Dentistry, University of São Paulo, Av. Professor Lineu Prestes, 2227, São Paulo 05508-000, Brazil; igorsm@usp.br; 9 Unit for Multidisciplinary Research in Biomedicine (UMIB), Institute of Biomedical Sciences Abel Salazar, University of Porto (ICBAS/UP), Porto 4050-313, Portugal

**Keywords:** resin-based dental composite, strength, mechanical properties, water uptake, BPA

## Abstract

(1) Background: The work aims to determine different chemical and mechanical properties with and without BPA dental resin–matrix composites under the same curing and testing conditions. (2) Methods: Disc-shaped specimens were prepared from six resin–matrix composites used in dentistry, three with BPA (BE-Brilliant EverGlow^TM^, ED-IPS Empress Direct, FS-Filtek^TM^ Supreme XTE) and three without (AF-Admira Fusion, BF-Enamel Plus HRi Bio Function Enamel, N/C). Specimens were photoactivated using an LED light-curing unit. The chemical and mechanical properties were analysed. (3) Results: The FS group exhibited the most significant water sorption (31.17 µg/mm^3^), while the BF showed the lowest (12.23 µg/mm^3^). Regarding the diffusion coefficient, the result recorded for the group AF is faster-absorbing water, and the group NC is slower. In both test methods (biaxial flexural strength and compressive strength), the resistance to flexural loading of the AF group was significantly lower than all other resin composites evaluated. (4) Conclusions: According to all the parameters studied, we verified that the BF presents the best chemical–mechanical behaviour. Resins free of BPA may not influence chemical–mechanical performance. However, the inorganic matrix has more influence on mechanical properties than the organic matrix.

## 1. Introduction

Dental resin has changed dental care by enabling minimally invasive procedures that maintain healthy tooth structures and provide natural-looking results [1]. Since the 1960s, resins have served as effective filling and restorative materials, improving the strength and appearance of dental work while being less harmful to health and the environment [2]. Their usage has increased, especially after the World Health Organization’s call to phase out dental amalgam [3]. Overall, dental resins have shown effective results.

The more conservative approach is to use dental resin in aesthetic and functional rehabilitation in young patients with dentition conditions, such as closing the spaces in maxillary lateral incisors agenesis [4]. The esthetic and functional restorations in the oral environment are subjected to different occlusal forces [5,6,7]. BPA is an organic compound commonly used in the manufacturing of polycarbonate plastics. These plastics are often found in items that come into contact with food, such as baby bottles and food containers. BPA is also utilised in the production of epoxy resins, which serve as protective coatings for canned food and beverages [8]. This causes the consumers to be exposed to BPA by ingesting food stored in these containers [9], which can eventually lead to localised and systemic toxicity [6,8,9,10] by ingesting and absorbing BPA via the oral and gastrointestinal mucosa.

BPA and its derivatives have also been identified as endocrine disruptors that effectively bind to and activate the oestrogen receptor [6]. In vivo studies have shown that administering BPA in low amounts has implications for both male and female reproductive systems [11]. Additionally, it may induce adverse effects in the brain, cardiovascular system, thyroid, intestines, and prostate [6].

The chemical composition of resin–matrix composites varies according to their clinical applications and manufacturers. The organic matrix often includes BPA monomers and derivatives such as Bis-GMA (bisphenol A-glycidyl methacrylate), Bis-DMA (bisphenol A-dimethacrylate), Bis-EMA (ethoxylated bisphenol A methacrylate), Bis-PMA (propoxylated bisphenol A-dimethacrylate), BADGE (bisphenol A diglycidyl ether), PC Bis-GMA (polycarbonate-modified Bis-GMA), and Bis-MPEPP (bisphenol A polyethoxy methacrylate) [12]. TEGDMA (triethylene glycol dimethacrylate), UDMA (urethane dimethacrylate), and photoinitiators can also be present in the organic matrix composition. The inorganic content can reach up to 90 wt% of the resin–matrix composite, incorporating one or two types of silanised ceramic or glass–ceramic fillers such as colloidal silica, zirconium silicate, barium silicate, or ytterbium fluoride [2,13,14,15]. The balance in the percentages of the organic matrix and inorganic fillers determines the physicochemical properties of the resin–matrix composites [13,14,16,17,18]. Indeed, the resin–matrix links charges, affecting the thermal expansion coefficient, shrinkage, water absorption, and solubility [19].

BPA is not typically used in formulating composites. Although it is not a by-product of the degradation of Bis-GMA, it can be generated through the hydrolysis of other dimethacrylates [20]. When resin composites contain BPA impurities, these impurities can be released into an aqueous environment, leading to patient exposure to BPA [20]. Ideally, resin composites should not release BPA and should not pose a risk of BPA exposure. This can be achieved by utilising high-purity monomers, even if they come at a higher cost. Since impurities often arise from synthesising monomers with a BPA core, such as Bis-GMA and Bis-EMA, an alternative approach would be to use monomers that do not have a BPA core [21].

Bis-GMA is the predominant monomer used in commercial dental composites. Its dominance is attributed to its low volumetric shrinkage, high reactivity, good mechanical properties, low volatility, and diffusivity into tissues [22]. However, it also has negative aspects, such as high hydrophilicity and viscosity [23].

To prepare Bis-GMA-free dental materials, UDMA, another common dimethacrylate monomer used in dentistry, has been considered [24]. Compared to Bis-GMA-based resin, UDMA-based resin has a significant drawback due to its higher volumetric contraction [20]. This greater contraction can create a larger gap between the tooth and the restoration, increasing the risk of secondary caries [25], staining [26], and potential loss or fracture of the restoration [27]. Consequently, alternative monomer compositions to Bis-GMA have been introduced to address this product’s limitations regarding durability and toxicity. One alternative is a hybrid organoceramic for methacrylate-based resin–matrix composites, known as ORganically MOdified CERamic (ORMOCER^®^, VOCO GmbH, Cuxhaven, Germany), which is an Organically Modified SILicate (ORMOSIL). One of the most exciting characteristics of ORMOCER^®^ is the combination of polysiloxane groups with photopolymerisable methacrylate groups that are covalently bonded to silica fillers [28,29,30]. The oxygen is replaced by organic groups, creating a three-dimensionally polymerised material with less organic matrix than conventional resin–matrix composites. This results in high biocompatibility due to the absence of residual monomers, reduced polymerisation shrinkage, high wear resistance, increased opacity, and improved handling characteristics [31,32,33,34].

The present study aimed to evaluate the chemical and mechanical properties of six different dental resin–matrix composites, including three containing BPA and three free of BPA. These composites varied in their organic matrices, filler loading, and types of fillers, and all were tested under the same curing and testing conditions. The null hypothesis stated that there would be no significant differences in the following properties: (1) water sorption, solubility, and coefficient of diffusion, and (2) compressive strength, strain, Young’s modulus, and biaxial flexural strength, regardless of the presence or absence of BPA among the resin matrix composites.

## 2. Materials and Methods

### 2.1. Preparation of Specimens

The six resin–matrix composites tested in this study (Table 1 and Table 2) were as follows: Admira Fusion (VOCO, Cuxhaven, Germany), Enamel Plus HRi Bio Function Enamel (Micerium SpA, Avegno, Italy), N/C (Coltène-Whaledent, Altstätten, Switzerland), Brilliant EverGlow™ (Coltène-Whaledent, Altstätten, Switzerland), IPS Empress Direct (Ivoclar Vivadent, Liechtenstein), and Filtek™ Supreme XTE (3M GmbH, Seefeld, Germany).

**Figure 1:**
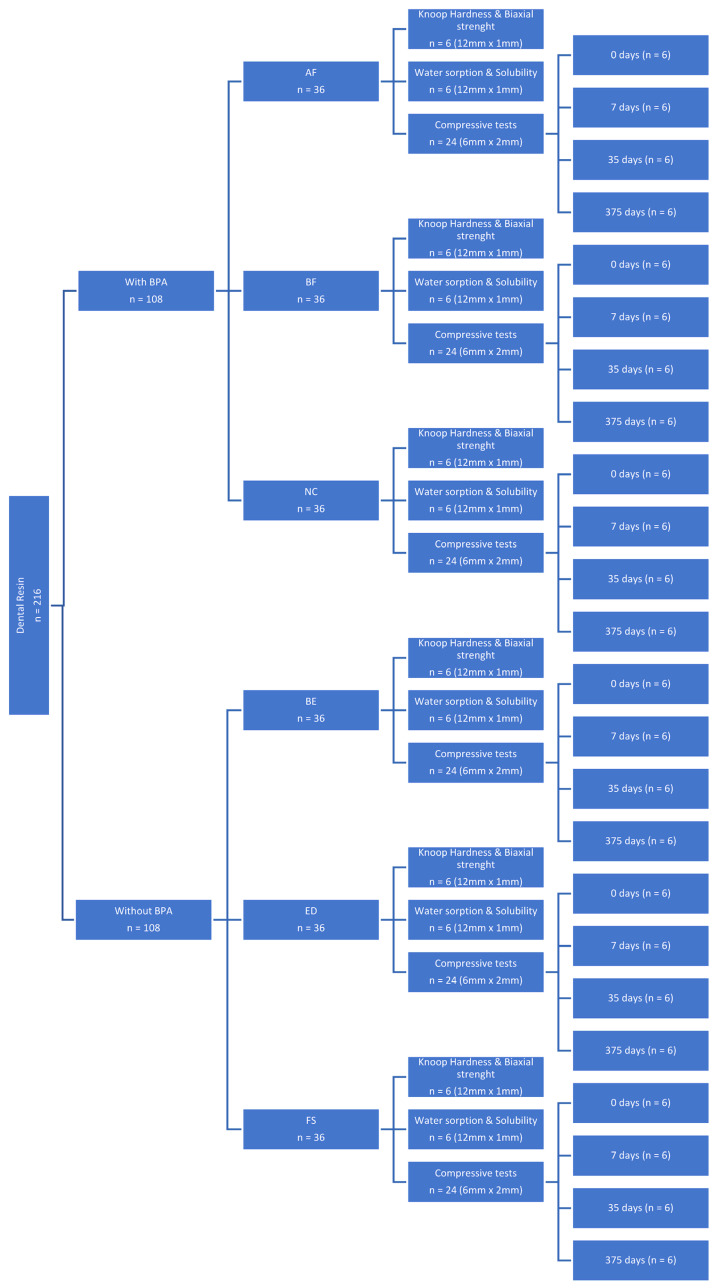
Flow chart of the research process with the number and dimension of the sample.

While condensing, the unpolymerised composite was placed in a stainless-steel mould, and a Mylar matrix strip was applied to the surface to limit oxygen inhibition. Excess material was removed using a sterile scalpel. Polymerisation was achieved with an LED light source (Celalux, High-Power LED curing light; VOCO, Cuxhaven, Germany) at an average intensity of 1300 mW/cm^2^ and wavelengths of 450/480 nm for 20 s on each side. Before each polymerisation, the equipment’s power was verified using a radiometer (Celalux, High-Power LED curing light; VOCO, Cuxhaven, Germany). After the light-curing process, the specimens were polished for 20 s using medium, fine, and super-fine discs (Sof-Lex, 3M ESPE, New York, NY, USA). The polishing discs were discarded after each polishing procedure, resulting in 36 samples for each resin, for a total of 216 (Figure 1). The specimens were stored in distilled water at 37 °C in the dark for 24 h immediately after curing.

### 2.2. Test of Specimens

Different tests were performed to evaluate stiffness as a function of the side that will be exposed to irradiation, to induce the reticulation of the resin through Knoop hardness tests, and to determine the effect of ageing on the compression strength of resins.

#### 2.2.1. Knoop Hardness and Biaxial Strength

Hardness values of brittle materials typically rise as indentation forces diminish, a phenomenon referred to as the indentation size effect (ISE) [35]. Due to the ISE, it is essential to establish a specified force or functional relationship over a test range to compare the hardness of brittle dental materials accurately.

Six samples (n = 6) of each investigated material were prepared with a diameter of 12 mm and a height of 1 mm. The sampler (Styleitaliano^TM^ products) allows the production of composite disc specimens with the desired thickness in cylindrical moulds: 12 ± 0.2 mm in diameter and 1 ± 0.1 mm in thickness. Visible light was irradiated for 20 s, and all specimens were entirely covered with transparent plastic matrix strips during this time.

The Knoop microhardness of the irradiated side and the bottom side of the hardened resin composite were then measured with Hardness Tester (Model no. HMV-2; Shimadzu, Kyoto, Japan). The specimens were positioned centrally below the diamond indenter to measure the Knoop hardness number (*KHN*) (Equation (1)).

(1) $$KHN=14.2\times \left(\frac{F}{d^{2}}\right)$$

The hardness of each specimen was measured with a 50 g load, applied for 15 s. The *KHN* equivalent to each indentation was calculated by computing the indentation dimensions and using the formula where *F* represents the test load and *d* refers to the longer diagonal of an indentation.

#### 2.2.2. Water Sorption and Solubility

**Figure 2:**
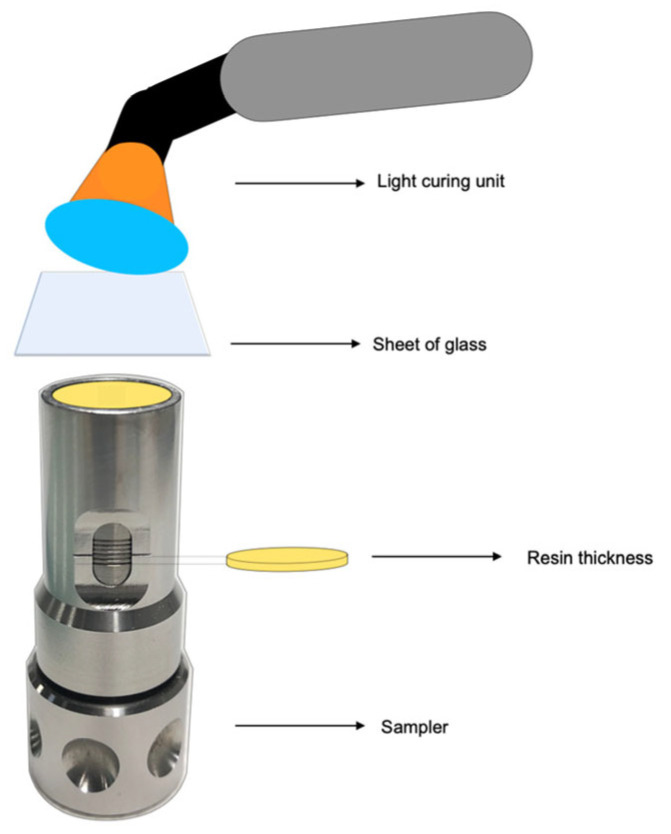
Production process of the discs. Sampler resin application, a glass sheet and light-curing, removal from the sampler, and light-curing and light-curing opposite sides.

Water sorption and solubility tests were conducted according to the composite specification standard (ISO 4049:2009) [36]. Six specimens were prepared for each testing condition. The specimens, with a 12 ± 0.2 mm diameter and a 1 ± 0.1 mm thickness, were fabricated using the sampler described above (Figure 2).

The thickness and diameter of the disks were measured using a digital electronic caliper (Mitutoyo Corporation, Tokyo, Japan). Each composite material specimen was prepared and weighed on an analytical scale accurate to 0.0001 mg (Sartorius, Bl210s, Göttingen, Germany). Before water immersion, the specimens were completely dried. All specimens were kept in a vacuum desiccator at 37 °C until a constant weight was achieved. At this stage, it was assumed that no water remained on the specimens (*M*_1_). The samples were then stored in 10 mL of distilled water. The mass of the specimens was periodically monitored until a stable value was recorded. At this stage, it was considered that the specimens were saturated. After this storage period, the samples were removed. Excess surface water was removed with absorbent paper until no water was visible, ensuring that the mass measured was contained within the specimen and not left on its surface. The weights were then re-recorded (*M*_2_). Subsequently, the specimens were reinserted into the desiccator at 37 °C and weighed daily until a constant mass was achieved (*M*_3_). At this stage, it was concluded that the specimen had dried again.

Equations (2) and (3) were used to calculate the water sorption (*WS*) and solubility (*SL*) values, respectively.

(2) $$WS=\frac{\left(M_{2}-M_{1}\right)}{M_{1}}\times 100$$

(3) $$SL=\frac{\left(M_{1}-M_{3}\right)}{M_{1}}\times 100$$

M_1_ is the initially dry specimen’s constant mass, in micrograms (mg), before water immersion, *M_2_* is the mass of the specimen (mg) after immersion in water for seven days; *M_3_* is the mass of the reconditioned specimen (mg), and *V* is the volume of specimen, in cubic millimetres (mm^3^).

Fick’s law of diffusion, given by Equation (4) [37], can be fitted to the specimen’s sorption and desorption behaviour.

(4) $$M_{t}=\left[1-\frac{8}{\pi^{2}}\sum_{n=0}^{\infty }\frac{1}{{\left(2n+1\right)}^{2}}exp\left(\frac{-D{\left(2n+1\right)}^{2}\pi^{2}t}{{4h}^{2}}\right)\right]M_{\infty }$$

where *t* represents the time starting from the immersion, and *h* represents the thickness of the specimen.

The water uptake at the time increment *t*, *M_t_*, is given by Equation (5).

(5) $$M_{t}=\frac{M}{M_{0}}$$

where *M* is the mass of the specimen at the time increment *t*, and *M*_0_ is the reference mass of the specimen at the beginning of the process.

A script was run in MATLAB (vR2023a, MathWorks) to determine the best fit of the coefficient of diffusion, *D*, and infinite water uptake, *M_t_*, to the experimental results.

#### 2.2.3. Compressive Tests Procedure

**Figure 3:**
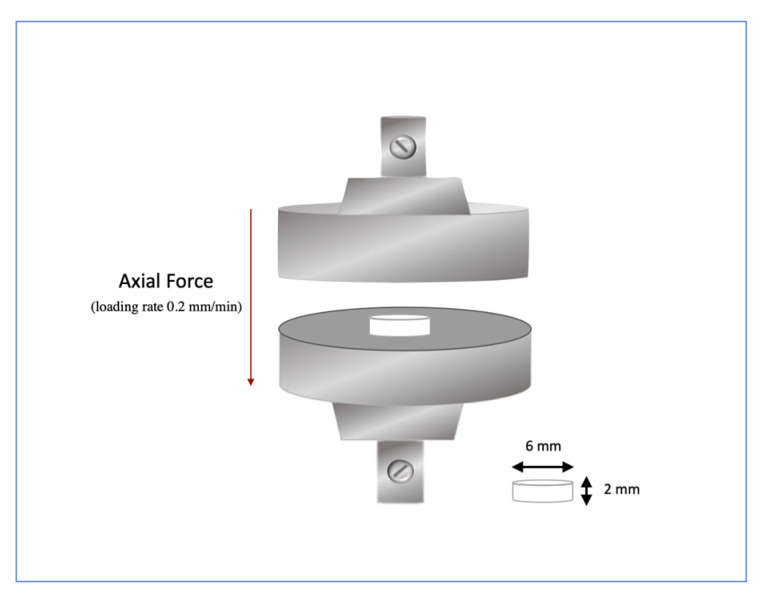
Compressive test.

Compressive strength is defined as the stress at which it fractures. The tests were conducted using a universal testing machine, INSTRON^®^ model 3367 (INSTRON, Norwood, MA USA), equipped with a 30 kN load cell and set at a displacement rate of 0.2 mm/min (Figure 3). First, load and strain were recorded electronically using the TestXpert II software (vV3.2, Zwick GmbH & Co. KG, Ulm, Germany). Afterwards, the recorded data were converted into the mechanical parameters: Young’s modulus and compressive strength.

Twenty-four samples (n = 24) of each investigated material were prepared with a diameter of 6 mm and a height of 2 mm (Figure 3). All specimens were placed in a vacuum desiccator and stored in the oven at 37 °C to ensure uniform conditions. Next, the specimens were stored in 10 mL of distilled water for seven days, 36 days, and one year. After this storage period, the samples were removed, and excess water was blotted away with absorbent paper until no water was visible. Finally, a compressive test was conducted on the specimens after preparation (0 days) and after 7 days, 36 days, and one year of immersion in distilled water.

Compressive strength (*CS*) was calculated from Equation (6):

(6) $$CS=\frac{4F}{{\pi d}^{2}}$$

*F* is the maximum load, and *d* is the cylindrical specimen diameter.

### 2.3. Statistical Analysis

The Statistical Package for Social Sciences (SPSS) Version 25 program from International Business Machines (IBM, Armonk, NY, USA) was used for the statistical analysis. The normality of data distribution was assessed using the Shapiro–Wilk test, and the homogeneity of variances was assessed using the Levene test. The differences between the mean values of the Knoop hardness, water sorption and compressive strength tests were analysed using a one-way analysis of variance (ANOVA), followed by Tukey’s post hoc test for multiple comparisons. Statistical significance was set at a level of *p* < 0.05.

## 3. Results

### 3.1. Knoop Hardness and Biaxial Strength

**Figure 4:**
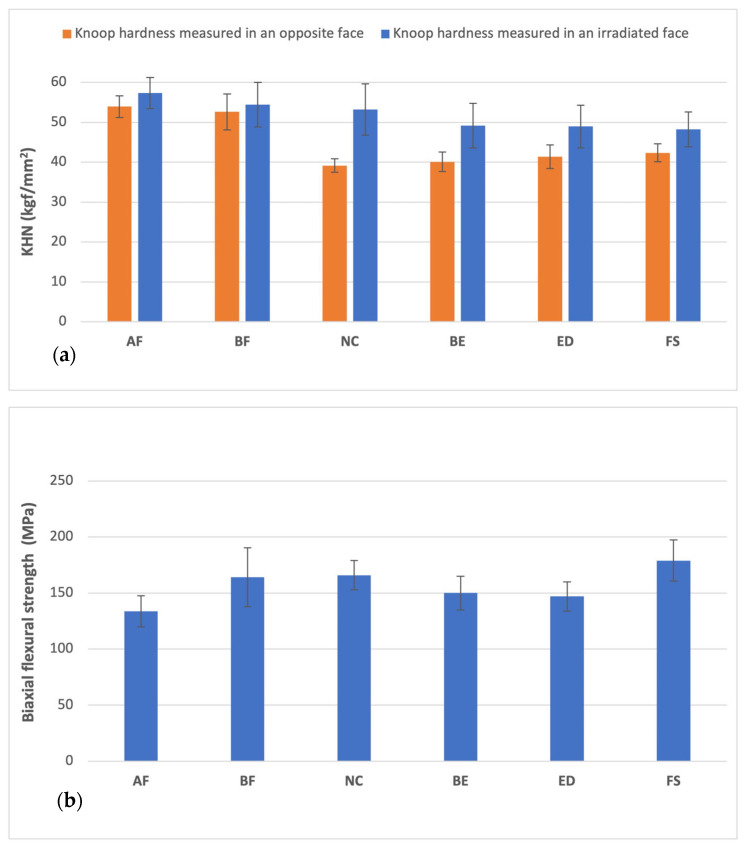
Material properties of the resin composite specimens: (**a**) Knoop hardness (Kgf/mm^2^), (**b**) biaxial flexural strength (MPa) (*p* < 0.05).

Knoop hardness values on the irradiated surface were statistically superior to those obtained on the opposite surface (Figure 4a and Table 3). The AF group shows the highest Knoop hardness values (57.31 ± 3.88), and the FS group shows the lowest value (48.19 ± 4.37) (Table 3). The Knoop hardness is higher in the resin group without BPA than with BPA (AF > BF > NC > BE > ED > FS).

As shown in Figure 4b and Table 4, the biaxial flexural strength results recorded for the BE and ED groups were quite similar (BE: 150.0 ± 15.00 and ED: 146.9 ± 12.90). Moreover, the AF group showed the lowest value (133.7 ± 13.90), and the FS group was the highest (178.9 ± 18.50).

Although the AF group showed the highest hardness value, it had the lowest flexural strength. On the other hand, the FS group was the soft material but with the highest flexural strength. Therefore, considering the hardness and flexural strength, the BF and NC groups were the ideal materials for the scope of dental resin. They were hard but showed moderate flexibility to avoid the material’s fracture.

### 3.2. Water Sorption and Solubility

The FS group exhibited the highest water sorption (31.17 µg/mm^3^), while the BF group showed the lowest (12.23 µg/mm^3^), as indicated in Table 5. There were no significant changes in the materials’ water sorption values between 7 days and 36 days of immersion (Table 5). The AF group maintained a constant water sorption value of 18 µg/mm^3^. After 20 days of storage, FS demonstrated the highest water sorption (33.69 µg/mm^3^). The BF group is the most hydrophobic resin, displaying the lowest water sorption (Table 5). The water sorption values across different groups fall within the ISO standard range [36] (Wsp < 40 µg/mm^3^). Water solubility also varied among the groups (Table 6). FS had the lowest mean water solubility value, followed by NC. In contrast, ED and AF exhibited the highest solubility values. The water solubility values for the different groups are significantly below the ISO guidelines [36] (Wsl < 7.5 µg/mm^3^).

**Figure 5:**
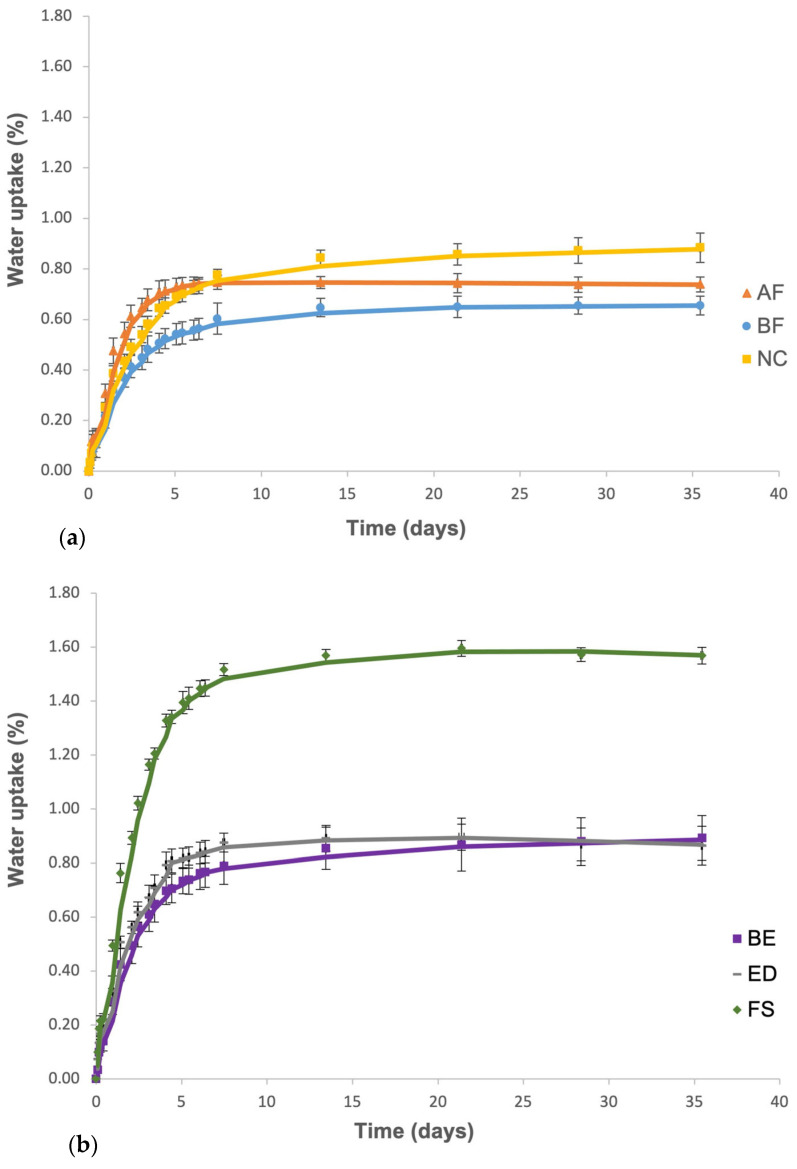
Experimental results and analytical model of the gravimetric test of resin groups: (**a**) without BPA (AF, BF, NC); (**b**) with BPA (BE, ED, FS) (*p* < 0.05).

The results obtained from Fick’s law of diffusion (Figure 5) indicate that the FS group (nanofilled resin) absorbs more water (1.6%) in the first seven days than the other groups (nanohybrid resins). However, after seven days, the water uptake rate for all groups decreases until it reaches a constant value. The amount of water a resin–matrix composite can absorb is influenced by the polymeric matrix’s hydrophilicity and the filler’s composition. This may be due to reduced sites where water molecules can form hydrogen bonds with the material, as these bonds are established between the matrix and water. Additionally, the material has lower porosity because part of it is occupied by inorganic filler, which decreases the water absorbed into the free volume of the polymeric chain within the resin matrix. Regarding the diffusion coefficient (*D*), group AF is the fastest in absorbing water, while group NC is the slowest (Table 7).

### 3.3. Compressive Tests

**Figure 6:**
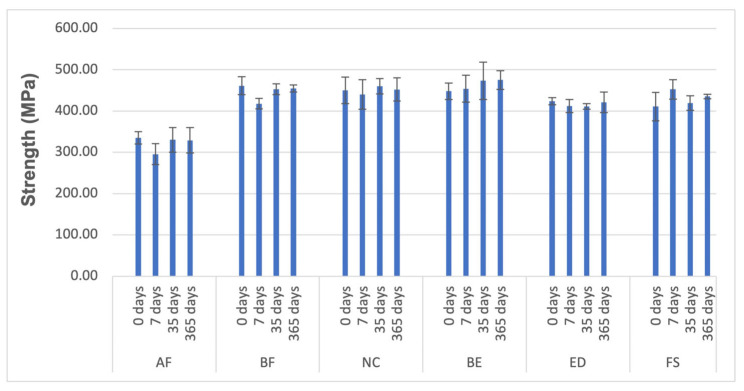
Material properties of the resin composite specimens: Strength (MPa), *p* < 0.05.

**Figure 7:**
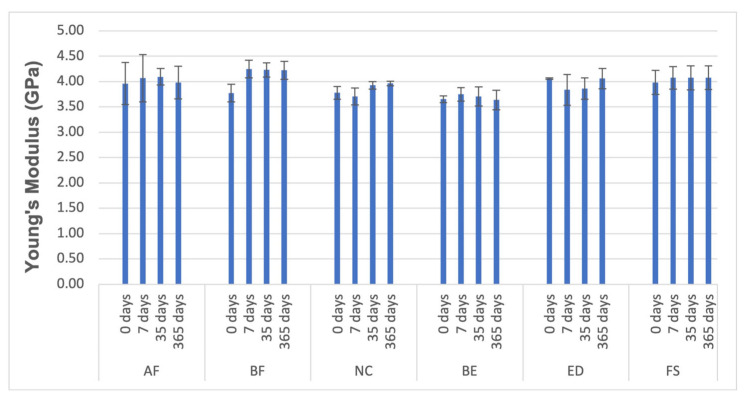
Material properties of the resin composite specimens: Young’s modulus (GPa), *p* < 0.05.

**Figure 8:**
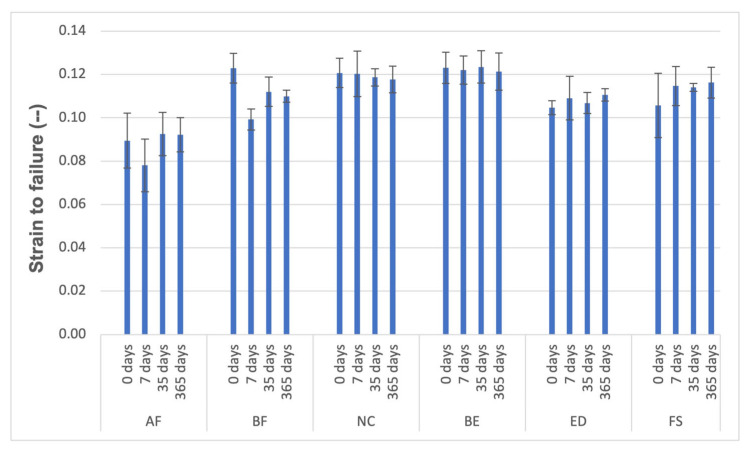
Material properties of the resin composite specimens: Strain to failure (-), *p* < 0.05.

The results of the mechanical properties assessed from the compressive tests are presented in Figure 6, Figure 7 and Figure 8 and Table 8, Table 9 and Table 10. Regarding flexural strength (FS), after 7 days of wet storage, all groups were statistically comparable except for AF and BF, which demonstrated a decrease in strength, while FS displayed an increase in strength (Table 8). After one year of hydration, the materials exhibited the same behaviour they showed before water immersion (0 days). According to the conducted tests, the effect of diffusion on the materials’ behaviour in compressive tests is nearly negligible.

The statistical analysis results of biaxial flexural strength and compressive strength are displayed in Figure 4b and Figure 6. In both testing methods, the AF group exhibited significantly lower resistance to flexural loading compared to all other evaluated resin composites. Additionally, the biaxial test method reached the same conclusions (FS>BF>NC>BE>ED>AF), except for the BE group, which showed a lower outcome compared to the compressive test (BE>FS>NC>BF>ED>AF).

## 4. Discussion

Surface hardness has been used as an indicator for monomer conversion [38,39,40,41,42]. Knoop hardness values on the irradiated surface were statistically superior to those obtained on the opposite surface (Figure 4a and Table 3). Therefore, the irradiated surface is the face where monomer conversion is most effective, and hardness is higher in the irradiated face. A low hardness value of a resin composite indicates poor chemical/physical bonding between the matrix and filler interface [39,42,43]. The hardness of composites has been positively correlated with how the organic matrix is formed [44] and filler weight % [37,42,43,45] (Figure 4b and Table 2), as evidenced in this study (AF>BF> BE = ED>FS). Therefore, we opted to evaluate the compression tests on the irradiated face of the resin discs.

Materials made from hydrophilic monomers, such as resin–matrix composites, have high water sorption, as expected. The water sorption (WS) of dental materials plays a crucial role in their long-term stability in aqueous environments, such as the mouth, and could be affected by dietary erosive factors [46]. Water intrusion can lead to several adverse effects, including the hydrolysis of the polymeric network [47], reduced thermal stability [48], compromised mechanical properties [49], and the leaching of unreacted monomers [50]. Additionally, water solubility (WSL) indicates the amount of unreacted monomers released from polymer networks. The release of these monomers is a primary source of cytotoxicity and can result in tissue inflammation [51]. For these reasons, evaluating the WS and WSL of resin–matrix composites under in vitro conditions with distilled water is essential. The results will be more effective than in saliva, where other substances may serve as a barrier to water absorption by the material. The FS group exhibited the highest water sorption (31.17 µg/mm^3^), while the BF group showed the lowest (12.23 µg/mm^3^), as shown in Table 5. These results are consistent with the reports by Kumar and Sangi [37], which indicated that the nanofilled composite (FS group) has higher water sorption and solubility, while the nanohybrid resin-like groups (AF, BF, NC, BE, and ED groups) have lower water sorption and solubility. It could be explained by the porous nature of nanoclusters, which makes them more prone to ion leaching and hydrolysis of the silane coupling agent, leading to the detachment and loss of filler particles. Dental composites absorb water, slowly accumulating between the inorganic fillers and matrix. This process can take up to 2 months to reach equilibrium [52,53,54]. Variations in water sorption were noted between 7 days and 36 days of immersion (Table 5). The water uptake of fluorinated resins was significantly lower than that of Bis- GMA- based resins [55]. Consequently, the lower WS and water uptake observed in the BF group may be attributed to the fluorinated structure within its filler composition. With its reduced WS, the BF resin may demonstrate superior water resistance compared to the Bis-GMA-based resin.

By incorporating fluorine into the UDMA monomer backbone, we can significantly enhance mechanical properties [14,56]. Stansbury JW et al. have suggested that fluorinated urethane dimethacrylate (FUDMA) could be a reliable alternative to Bis-GMA in dental materials. Its exceptional durability, resistance to a wide range of solvents, and hydrophobicity make it a reassuring choice. The strong chemical bond between fluorine and carbon gives fluoro-polymers excellent chemical resistance to water, instilling confidence in their performance in various conditions [14,51].

Aesthetic and functional dental restorations are subjected to various occlusal forces influenced by age, gender, and body mass index. More research is needed on materials that can handle higher loads. Dental composites may face excessive forces from injuries in traffic accidents or sports, leading to fractures in both the tooth and the restoration.

A biomimetic approach advocates for resin–matrix composites with mechanical properties resembling enamel, dentin, and enamel–dentin, including compressive strength, strain-to-failure, and elastic Young’s modulus (E). The strengths of the enamel, dentin, and enamel–dentin specimens were 62.2 ± 23.8, 193.7 ± 30.6, and 126.1 ± 54.6 MPa, respectively. The Young’s moduli of the enamel, dentin, and enamel–dentin specimens were 1338.2 ± 307.9, 1653.7 ± 277.9, and 1628.6 ± 482.7 MPa, respectively. The strain-to-failure values for the enamel, dentin, and enamel–dentin specimens were 4.5 ± 0.8%, 11.9 ± 0.1%, and 8.7 ± 2.7%, respectively [57]. Compared to our results, the behaviour of the materials studied in terms of strengths and Young’s moduli was superior. In contrast, the strain to failure was lower than enamel and dentin.

The stress–strain curves for the tested materials are derived from measured values, which help determine Young’s modulus. There are differences in the composition and matrix ratios of the materials examined; consequently, their responses to dynamic loading vary. Based on the stress–strain curves, we calculated the dynamic elastic moduli of the tested materials. The flexural strength simulates using composites in areas with high stress and indicates the material’s resistance to fracture. In addition, there is an ISO 4049:2009, with an 80 MPa minimum limit for polymer-based restorative materials, which manufacturers have stated is suitable for occlusal surfaces [36]. Below that threshold, composites would be theoretically vulnerable to early fracture in vivo. Different groups’ mechanical test values (Knoop hardness, biaxial flexural strength, and compressive test) are within the range of ISO standards. The FS group (nanocharged resin) exhibits better compressive strength due to the closer interparticle distance between the nanoparticles, which reduces crack formation and propagation. The smooth, rounded edges of the nanoparticles also help distribute stress evenly throughout the composite resin, aligning with findings from De Moraes et al. [58].

According to Hooke’s law, the elastic modulus (*E*), or Young’s modulus, is the ratio of stress to strain within the material’s elastic range. It indicates the material’s ability to store elastic energy associated with recoverable elastic deformation. A stiffer material has a higher Young’s modulus, meaning it experiences less elastic strain when stress is applied. The loss modulus (*E′*) reflects the material’s capacity to dissipate mechanical energy by converting it into heat through molecular motion, corresponding to unrecoverable viscous loss. High values were reported for the nanohybrid BF group, while the lowest were noted for the submicron hybrid BE group (Figure 7). The BF was identified as the stiffest composite tested. It is according to Luo S et al. who found that FUDMA-based composites demonstrate higher flexural strength and modulus compared to Bis-GMA and UDMA-based composites, attributed to their higher DC and the presence of aliphatic rings and a fluorinated chain—(CF2)6—that is believed to induce rigid crosslinking of the polymer [59].

The significance of the elastic modulus is primarily related to selecting an appropriate composite for a specific clinical situation. For instance, flowable composites are typically utilised in V-class restorations because their higher elasticity can better absorb chewing forces in this tooth region.

Hydrolytic degradation is a rate-dependent diffusion process influenced by the type of polymer, the type of filler particles, and the surface treatment of the filler particles. In this study, water ageing did not cause a significant reduction in strength after immersion. When the matrix becomes saturated with water and softens, the composite structure stabilises, and no further decrease in properties occurs within the studied period. Carreiro et al. [60] conducted a similar experiment in which they immersed different resin composite materials in distilled water for 180 days to test their compressive strengths. They found that the compressive performance of the same composite material remained relatively unchanged. The variations in compressive strength among different composite materials may be linked to factors such as load volume and the specific resin formulation used. AF and ED resins, which contained barium glass (Table 2), showed decreased flexural strength (Figure 6 and Figure 8). Barium silicate glass may be linked to this reduced flexural strength, as it was found that when exposed to water, barium was exchanged with the hydrogen ions present in water, making barium silicate glass sensitive to water [61].

This study has some limitations due to the diverse compositions of the materials studied, which extended beyond the presence or absence of BPA. To address this, it is essential to design a study that applies the same methodology to groups of resins with identical compositions, differing only in the presence or absence of BPA. The optimal approach would be to conduct a study with two groups of resins with the same composition, where the sole difference is the absence of BPA. This could significantly influence future studies by providing a more controlled investigation of BPA’s effects.

In summary, we confirmed that the BF group exhibits the best overall chemical and mechanical behaviour based on all the parameters studied. This BPA-free nanohybrid resin–matrix composite absorbs less water, demonstrates high strength, and offers some flexural resistance compared to the other groups.

## 5. Conclusions

Within the limitations of this study, the following conclusions were drawn:

1. No significant differences exist between groups of resins containing BPA and those free from this monomer and its derivatives. Therefore, the absence of BPA in resins may not affect the chemical–mechanical performance of composite resins.
2. The AF group is a promising Bis-GMA-free and Ormocer^®^-based material; however, it does not perform mechanically comparably to conventional Bis-GMA-containing resin–matrix composites. Additionally, AF seems more prone to mechanical degradation effects caused by water, particularly in the early stages of diffusion. The inorganic matrix may exert more influence than the organic matrix regarding the studied parameters, where BPA and its derivatives are in their composition.

In the future, the goal would be to conduct a study using the same methodology, yet with two groups of resins featuring the same composition, the only difference being the absence of BPA.

## Figures and Tables

**Table 1:** Materials used in this study and manufacturers’ information.

Group	Brand	Code	Lot number	Classification
AF	Admira^®^ Fusion	A2 enamel	19076721	Nanohybrid-ORMOCER
BF	Enamel Plus HRI BIO Function	A2 enamel	2018003080	Nanohybrid
NC	Experimental resin	----	C1971	Nanohybrid
BE	BRILLIANT EverGlowTM	A2/B2	J18375	Submicron hybrid
ED	IPS Empress Direct	A2 enamel	Y28474	Nanohybrid
FS	FiltekTM Supreme XTE	CT enamel	NA28205	Nanofilled

**Table 2:** Materials used in this study, their composition, and manufacturers’ data.

	Group	Organic Matrix	Filler	Filler by Weight (%)	Filler Dimension (μm)	Water Sorption (μg/mm^3^)	Water Solubility (μg/mm^3^)
Without BPA	AF	ORMOCER^®^ resin	SiO_2_ Ba-Al-B-Si-glass fillers	84	2.5 to 3.0	13.4	≤0.1
	BF	UDMA, TCDDMA, no co monomers, and no Bis-GMA	Glass filler, high dispersion silicon dioxide, fluorine	74	0.2 to 3.0	15.27	0.31
	NC	Not available	Barium glass submicron	Not available	Not available	Not available	Not available
With BPA	BE	Bis-GMA TEGDMA Bis-EMA	ZnO Amorphous silica sillers	79	0.4 to 0.7	15.1	<0.1
	ED	Bis-GMA UDMA TCDD	Ba-Al-Si-glass YbF3, SiO_2_/ZrO_2_, MO, Nanomodifier	78	0.1 to 0.3	19.6	<0.1
	FS	Bis-GMA UDMA TEGDMA Bis-EMA	ZrO_2_/SiO_2_ cluster SiO_2_ nano-scale fillers	72.5	0.6 to 20	Not available	Not available

Abbreviations—BPA: Bisphenol A; Bis-GMA: bisphenol A diglycidyl methacrylate; Bis-EMA: ethoxylated bisphenol A dimethacrylate; TEGDMA: triethylene glycol dimethacrylate; UDMA: urethane dimethacrylate; TCDDMA: tricyclodecane dimethanol dimethacrylate; TCDD: 2,3,7,8-tetrachlorodibenzo-p-dioxin.

**Table 3:** Mean, SD values, and coefficient of Knoop hardness for all groups (*p* < 0.05).

Material	Irradiated Face		Opposite Face	
	Knoop Hardness (Mean ± SD)	Coefficient (%)	Knoop Hardness (Mean ± SD)	Coefficient (%)
AF	57.31 ± 3.88	7	53.92 ± 2.75	5
BF	54.44 ± 5.62	10	52.60 ± 4.53	9
NC	53.18 ± 6.42	12	39.13 ± 1.70	4
BE	49.18 ± 5.60	11	40.08 ± 2.43	6
ED	48.95 ± 5.36	11	41.36 ± 2.99	7
FS	48.19 ± 4.37	9	42.31 ± 2.26	5

**Table 4:** Mean, SD values, and coefficient of biaxial flexural strength (MPa) for all groups (*p* < 0.05).

Material	Flexural Strength [MPa]	Coefficient (%)
AF	133.7 ± 13.90	10
BF	164.0 ± 26.30	16
NC	165.9 ± 13.10	8
BE	150.0 ± 15.00	10
ED	146.9 ± 12.90	9
FS	178.9 ± 18.50	10

**Table 5:** Mean and SD water sorption values for all groups for a total immersion period of 7 and 36 days (*p* < 0.05).

Material	Water Sorption (µg/mm^3^)		
	Brand	7 Days	36 Days
AF	13.4	18.19 ± 0.93	18.16 ± 1.03
BF	15.27	12.23 ± 0.82	14.08 ± 0.75
NC	Not available	15.89 ± 0.83	19.16 ± 1.21
BE	15.1	15.80 ± 1.55	18.02 ± 0.76
ED	19.6	19.47 ± 0.82	20.05 ± 0.89
FS	Not available	31.17 ± 3.53	33.69 ± 4.23

**Table 6:** Mean and SD values of water solubility for all groups (*p* < 0.05).

Material	Water Solubility (µg/mm^3^)	
	Brand	Study
AF	≤0.1	2.29 ± 0.53
BF	0.31	1.55 ± 1.10
NC	Not available	1.28 ± 0.53
BE	<0.1	1.48 ± 0.75
ED	<0.1	3.48 ± 1.88
FS	Not available	1.11 ± 0.40

**Table 7:** Water uptake properties of the materials analysed.

Material	D (m^2^/s)	M∞ (%)
AF	9.8 × 10^−13^	0.63
BF	7.5 × 10^−13^	0.57
NC	3.8 × 10^−13^	0.84
BE	8.4 × 10^−13^	0.59
ED	9.3 × 10^−13^	0.83
FS	5.2 × 10^−13^	0.12

**Table 8:** Mean and SD values of strength, Young’s modulus, and strain failure for all groups for a total immersion period of 7 days, *p* < 0.05.

Material	Strength (MPa)	Young’s Modulus (GPa)	Strain to Failure (-)
AF	295.55 ± 25.45	4.07 ± 0.47	0.08 ± 0.01
BF	417.73 ± 12.81	4.25 ± 0.18	0.10 ± 0.00
NC	440.52 ± 35.82	3.71 ± 0.16	0.12 ± 0.01
BE	453.89 ± 32.73	3.75 ± 0.13	0.12 ± 0.01
ED	411.87 ± 16.04	3.84 ± 0.31	0.11 ± 0.01
FS	452.59 ± 23.41	4.07 ± 0.22	0.11 ± 0.01

**Table 9:** Mean and SD values of strength, Young’s modulus, and strain failure for all groups for a total immersion period of 36 days, *p* < 0.05.

Material	Strength (MPa)	Young’s Modulus (GPa)	Strain to Failure (-)
AF	330.11 ± 29.58	4.10 ± 0.17	0.09 ± 0.01
BF	452.98 ± 13.37	4.23 ± 0.14	0.11 ± 0.01
NC	460.05 ± 18.91	3.93 ± 0.08	0.12 ± 0.00
BE	473.33 ± 45.24	3.70 ± 0.19	0.12 ± 0.01
ED	411.10 ± 6.70	3.86 ± 0.21	0.11 ± 0.00
FS	419.10 ± 17.84	4.07 ± 0.24	0.11 ± 0.00

**Table 10:** Mean and SD values of strength, Young’s modulus, and strain failure for all groups for a total immersion period of 1 year, *p* < 0.05.

Material	Strength (MPa)	Young’s Modulus (GPa)	Strain to Failure (-)
AF	328.93 ± 30.65	3.98± 0.32	0.09 ± 0.01
BF	454.85 ± 8.73	4.22 ± 0.18	0.11 ± 0.00
NC	452.32 ± 28.46	3.96 ± 0.04	0.12 ± 0.01
BE	475.23 ± 22.82	3.64 ± 0.19	0.12 ± 0.01
ED	421.25 ± 24.70	4.06 ± 0.20	0.11 ± 0.00
FS	435.30 ± 5.41	4.07 ± 0.23	0.01

## Data Availability

The original contributions presented in this study are included in the article. Further inquiries can be directed to the corresponding author.

## Abbreviations

The following abbreviations are used in this manuscript:

BPA	bisphenol A
Bis-GMA	bisphenol A-diglycidyl methacrylate
Bis-DMA	bisphenol A-dimethacrylate
Bis-EMA	ethoxylated bisphenol A methacrylate
Bis-PMA	propoxylated bisphenol A-dimethacrylate
BADGE	bisphenol A diglycidyl ether
PC Bis-GMA	polycarbonate-modified Bis-GMA
Bis-MPEPP	bisphenol A polyethoxy methacrylate
TEGDMA	triethylene glycol dimethacrylate
UDMA	urethane dimethacrylate
ORMOCER	organically modified ceramic
FUDMA	fluorinated urethane dimethacrylate
AF	Admira^®^ Fusion
BF	Enamel Plus HRI BIO Function
NC	Experimental resin
BE	BRILLIANT EverGlow^TM^
ED	IPS Empress Direct
FS	Filtek^TM^ Supreme XTE
CS	compressive strength
WSL	water solubility
WS	water sorption
*E*	elastic modulus
*E′*	loss modulus
DC	degree conversion
